# Wavelet-Based Image Registration and Segmentation Framework for the Quantitative Evaluation of Hydrocephalus

**DOI:** 10.1155/2010/248393

**Published:** 2010-04-13

**Authors:** Fan Luo, Jeanette W. Evans, Norma C. Linney, Matthias H. Schmidt, Peter H. Gregson

**Affiliations:** ^1^Mathematics and Computing Science Department, Saint Mary's University, Halifax, NS, Canada B3H 3C3; ^2^Department of Psychiatry, University of British Columbia, Vancouver, BC, Canada V6T 2A1; ^3^Department of Radiology, Dalhousie University, Halifax, NS, Canada B3H 2Y9; ^4^Electrical & Computer Engineering, Faculty of Engineering, Dalhousie University, Halifax, NS, Canada B3J 1Z1

## Abstract

Hydrocephalus, characterized by increased fluid in the cerebral ventricles, is traditionally evaluated by a visual assessment of serial CT scans. The complex shape of the ventricular system makes accurate visual comparison of CT scans difficult. The current research developed a quantitative method to measure the change in cerebral ventricular volume over time. Key elements of the developed framework are: adaptive image registration based on mutual information and wavelet multiresolution analysis; adaptive segmentation with novel feature extraction based on the Dual-Tree Complex Wavelet Transform; volume calculation. The framework, when tested on physical phantoms, had an error of 2.3%. When validated on clinical cases, results showed that cases deemed to be normal/stable had a calculated volume change less than 5%. Those with progressive/treated hydrocephalus had a calculated change greater than 20%. These findings indicate that the framework is reasonable and has potential for development as a tool in the evaluation of hydrocephalus.

## 1. Introduction

Hydrocephalus results from excessive accumulation of cerebrospinal fluid, leading to enlargement of the cerebral ventricles. The condition is commonly evaluated by visual comparison of serial CT scans of the head. However, the complex shape of the ventricular system and the differences in the angulation of slices combined with slight differences in positioning of the head from one CT study to the next can make direct visual comparisons of serial imaging studies difficult and of limited accuracy. This makes the quantitative assessment of the volume change desirable. 

Earlier methods for quantitatively assessing ventricular volume have included the diagonal ventricular dimension [1], the frontal and occipital horn ratio [2], the ventricular-brain ratio [3], the Evans ratio [4], Huckman's measurement [5, 6], and the minimal lateral ventricular width [7], among others. The previous attempts to quantitatively assess ventricular volume have focused on linear, ratio, or surface area estimates of ventricular size, and as such, have been limited by the fact that they try to estimate volume (a 3-dimensional construct) using 1- or 2-dimensional measurements [8, 9]. In many cases the estimates are based solely on measurements taken from a single axial slice, and may leave potential volumetric changes in the 3rd or 4th ventricles unaccounted for [8, 9]. The previous techniques that have tried to assess volumetric changes 3dimensionally have been time consuming, limiting their clinical applicability [8, 9]. Furthermore, often measurements appropriate for adults are not appropriate for pediatric patients and vice versa [1, 2, 10].

This paper describes a novel framework to measure the change in the volume of the ventricles using CT scans taken at two separate times. The method involves registering the two CT image sequences to be compared, automatically segmenting the ventricles in all the image slices, and calculating a volume change from the results. The framework was validated and verified on both physical phantom models and clinical data.

Image registration is used to align the second set of CT images with the first, thus making the volume calculations consistent, reducing the error caused by the partial volume effect and improving the accuracy of the calculated change in volume. The differences in angulation of the slices combined with the slight differences in positioning of the head from one CT to the next is referred to in this paper as the displacement of the human head. A number of image registration techniques have been described previously, including landmark techniques [11]; point-based and thin-spline-based methods [12]; mutual information-based methods [13–15]. The current research required a rigid registration technique to compensate for the rigid displacement of the head between the CT scans, while maintaining the differences in ventricular volume and shape. Both in-plane and out-of-plane displacements needed to be considered. The developed framework includes an adaptive rigid registration method based on mutual information combined with image gradient information, and wavelet multiresolution analysis.

Image segmentation is the process of separating out mutually exclusive homogeneous regions of interest and in this research is used to isolate the ventricles in preparation for the volume calculation. In this paper, the focus is on a variation of the watershed automated segmentation method. The watershed method suffers from an oversegmentation problem, and a number of methods proposed in the literature to overcome the problem have had varying success. Soille [16] introduced the H-minima transform, which modifies the gradient surface, suppressing shallow minima. Shafarenko et al. [17] used a modified gradient map as the input for the watershed algorithm in randomly textured color images. O'Callaghan and Bull [18] proposed a two-stage method, which is capable of processing both textured and nontextured objects in a meaningful fashion. In the current research, the Dual-Tree Complex Wavelet Transform (DT-CWT) was used to detect the texture boundaries and a novel feature extraction method used to optimize the segmentation results.

Once the images are registered and the ventricles are segmented, the framework calculates the change in volume. To validate the method developed in this study, physical phantoms of the brain and cerebral ventricles were constructed, using agar and water to simulate brain tissue and cerebrospinal fluid, respectively. The volume of the phantom ventricles was measured directly and was then calculated using the method described in this paper. Clinical data with known outcomes were also used to validate the results.

**Figure 1:**
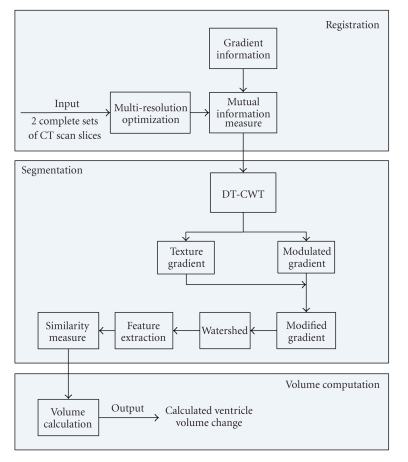
Algorithm framework.

In Section 2, Method, the registration method is described first, followed by the adaptive segmentation and feature extraction method and finally the volume calculation is discussed. The complete algorithm framework is shown in Figure 1. Section 3, Data Sets, describes the physical phantoms and the clinical data used to test the framework. Section 4, Results, summarizes and discusses the results. Conclusions are drawn in Section 5.

## 2. Method

### 2.1. Registration

The method described in this research uses an image registration technique to align the image slices of the CT scan taken at a time, *t*_2_, with the slices taken at an initial time, *t*_1_. This registration step reduces the error in the calculation of the volume change with time that would otherwise be caused by the partial volume effect [11]. In the following discussion *F*_*k*_(*x*_1_, *x*_2_) refers to the *k*th slice in the set of CT images, *F*_*k*_*t*_1___(*x*_1_, *x*_2_) refers to slice image *k* in the CT image scan taken at time *t*_1_, *F*_*k*_*t*_2___(*x*_1_, *x*_2_) refers to the closest corresponding CT slice image in the CT scan taken at the subsequent time *t*_2_, and ${\tilde{F}}_{k_{t_{2}}}(x_{1},x_{2})$ refers to image *F*_*k*_*t*_2___(*x*_1_, *x*_2_) after it has been registered to slice *F*_*k*_*t*_1___(*x*_1_, *x*_2_). *x*_1_, *x*_2_, *x*_3_ are the 3D spatial coordinates of the pixels and where *x*_3_ is not given, it is assumed to be in the image plane.

#### 2.1.1. Change in Volume Error

Given a clinical case with two different CT scans of the head taken at times *t*_1_ and *t*_2_, the cerebral ventricles will have a physical volume of *V*_*t*1_ and a calculated volume of *V*_*t*1_′ at time *t*_1_ and a physical volume of *V*_*t*2_ and a calculated volume of *V*_*t*2_′ at *t*_2_. Each calculated volume will have an error, *e*_1_ and *e*_2_, respectively, introduced in part by the partial volume effect, such that

(1) $$\begin{array}{c} V_{t1}^{\prime }=V_{t1}+e_{1},\\ V_{t2}^{\prime }=V_{t2}+e_{2}. \end{array}$$

Thus the change in calculated volume, Δ*V*′, between *t*_1_ and *t*_2_, is given by

(2) $$\begin{array}{c} \Delta V^{\prime }=V_{t2}^{\prime }-V_{t1}^{\prime }=V_{t2}-V_{t1}+\left(e_{2}-e_{1}\right). \end{array}$$

If the displacement of the head is such that the errors *e*_1_ and *e*_2_ compound, then Δ*V*′ will have a large error. If registration is applied so that the CT scans are aligned and the partial volume errors are consistent, then *e*_1_ will approach *e*_2_, and |*e*_2_ − *e*_1_| will approach zero.

If ${\tilde{V}}_{t2}^{\prime }$ represents the volume calculated using the set of registered images, ${\tilde{F}}_{k_{t_{2}}}\left(x_{1},x_{2}\right)$∀*k*, then

(3) $$\begin{array}{c} \Delta {\tilde{V}}^{\prime }={\tilde{V}}_{t2}^{\prime }-V_{t1}^{\prime }≃V_{t2}-V_{t1}=\Delta V. \end{array}$$

This means that if the set of images taken at *t*_2_ is registered to the set of images taken at *t*_1_, so that the partial volume errors are consistent, then the error in the calculated change in volume will be reduced. Since an accurate calculated change in volume is required for this work, the framework described in this research includes registration of the CT scans before the ventricles are segmented and their change in volume calculated.

#### 2.1.2. Modified Mutual Information

The registration method used in this research is a wavelet-based technique that maximizes the mutual information in the two image sets. The mutual information, *I*(*A*, *B*), of two images, *A* and *B*, is given by [13, 14, 19]

(4) $$\begin{array}{c} I\left(A,B\right)=H\left(A\right)+H\left(B\right)-H\left(A,B\right), \end{array}$$

where *H*(*A*) and *H*(*B*) are Shannon entropies for images *A* and *B*, respectively, and *H*(*A*,*B*) is the joint entropy between *A* and *B*. To reduce the effect of overlap, the more common form, normalized mutual information [20], *I*_*n*_(*A*, *B*), is used in this research

(5) $$\begin{array}{c} I_{n}\left(A,B\right)=\frac{H\left(A\right)+H\left(B\right)}{H\left(A,B\right)}. \end{array}$$

The entropies are computed by estimating the probability distributions of the image intensities. The joint entropy denotes the probability distribution of the image intensities shown in both the images *A* and *B*. 

The mutual information registration algorithm assumes that the images are geometrically aligned by the rigid transformation $T(\vec{\alpha })$, where $\vec{\alpha }$ is a vector consisting of six (three translation and three rotation) parameters. Optimal alignment is achieved with the set of parameters, $\vec{\alpha }={\vec{\alpha }}^{*}$, such that *I*_*n*_(*A*, *B*) is maximal. To achieve optimal alignment, the mutual information function must be smooth.

Because displacement of the human head between scans can be out-of-plane as well as in-plane, the framework in this research includes 3-dimensional registration using the complete set of image slices and trilinear interpolation. In order to reduce the local maxima effect, partial volume interpolation is used to provide a more accurate estimate of the joint histogram [21]. When the joint histogram is calculated for a subvoxel alignment, the contribution of the pixel intensity to the joint histogram is distributed over the intensity values of the eight nearest neighbours using weights calculated by trilinear interpolation.

To improve the performance and robustness of the mutual information measure used in the registration algorithm, it is combined with gradient information as outlined by Pluim et al. [22]. The method multiplies the mutual information with a gradient term that is based on both the magnitude and orientation of the gradients and is very briefly summarized here.

The gradient vector is computed for each sample point **x** = *x*_1_, *x*_2_, *x*_3_ in the reference image, *A*, which in this case is *F*_*t*_1__, and its corresponding point, $\tilde{x}$, in the registered image, *B* or ${\tilde{F}}_{t_{2}}$. $\tilde{x}$ is found using the rigid transformation, $T(\vec{\alpha })$, of **x**. The gradient terms are calculated by convolving the image with the appropriate first derivatives of a Gaussian kernel of scale *σ*. The angle $\alpha_{x,\tilde{x}}\left(\sigma \right)$ between gradient vectors is defined by

(6) $$\begin{array}{c} \alpha_{x,\tilde{x}}\left(\sigma \right)=\text{arccos}\frac{\nabla x\left(\sigma \right)*\nabla \tilde{x}\left(\sigma \right)}{\left|\nabla x\left(\sigma \right)\right|\left|\nabla \tilde{x}\left(\sigma \right)\right|} \end{array}$$

with ∇**x**(*σ*) denoting the gradient vector of scale *σ* at point **x**, |·| denoting its magnitude, and ∗ denoting the convolution operator. The gradient function, *G*(*A*, *B*), is computed as a weighted sum of the resulting products for all the pixels and is given by

(7) $$\begin{array}{cc} G\left(A,B\right) & =\underset{\left(x,\tilde{x}\right)\in \left(F_{t_{1}}\cap {\tilde{F}}_{t_{2}}\right)}{\sum }\omega \left(\alpha_{x,\tilde{x}}\left(\sigma \right)\right)\\ & \;\cdot \min\;\left(\left|\nabla x\left(\sigma \right)\right|,\left|\nabla \tilde{x}\left(\sigma \right)\right|\right), \end{array}$$

where the weighting function, $\omega (\alpha_{x,\tilde{x}}(\sigma ))$, smooths small angle variations and compensates for intensity inversions and is given by

(8) $$\begin{array}{c} \omega \left(\alpha_{x,\tilde{x}}\left(\sigma \right)\right)=\frac{\cos\;\left(2\alpha \right)+1}{2}. \end{array}$$

The new normalized mutual information *I*_*n*_′(*A*, *B*) becomes

(9) $$\begin{array}{c} I_{n}^{\prime }\left(A,B\right)=G\left(A,B\right)\cdot I_{n}\left(A,B\right). \end{array}$$

#### 2.1.3. Optimization Using Simplex Method and Multiresolution Decomposition

The six parameters in the registration function, $T(\vec{\alpha })$, are optimized simultaneously using the simplex method to find the global maximum. A drawback of this method is that if the mutual information function is not smooth with a single maximum, the simplex method may settle on a local maximum giving poor results. In order to reduce the impact of local maxima on the registration and improve the speed of the method the image resolution is reduced using a standard wavelet multi-resolution decomposition [23]. At the lower resolution, detail information is removed, the mutual information function is smoother, and local maxima are significantly suppressed. Also at the lower resolution only a fraction of the voxels in the image is used to construct the joint histograms so speed is improved. After the global maximum is found at the lower resolution, the resolution level is increased and initialization is based on the previously found maximum. Therefore, a combination of mutual information and multi-resolution analysis improves the chance of finding the global maxima in the mutual information function.

### 2.2. Adaptive Segmentation

An adaptive segmentation based on the watershed algorithm and a novel texture measurement is used in this research. The method consists of two stages: the preliminary watershed segmentation stage and the texture classification stage. In the first stage, DT-CWT coefficients are used to extract the texture gradient for the watershed algorithm. In the second stage, DT-CWT coefficients are used as the texture measure to classify the textures.

#### 2.2.1. Stage I: Modified Gradient for Preliminary Watershed Segmentation

**Figure 2:**
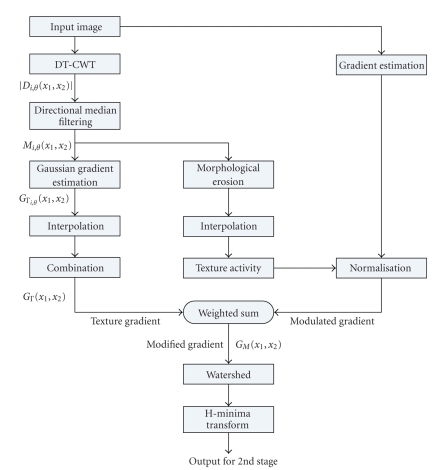
Segmentation algorithm: Stage I.

The first stage of the segmentation algorithm is outlined in Figure 2.

**(a) Texture Gradient**

The watershed algorithm is an automatic segmentation method based on visualizing a 2D image in 3-dimensions (two spatial dimensions, (*x*_1_, *x*_2_) and the image intensity, *F*(*x*_1_, *x*_2_)). Input to the watershed algorithm is gradient information from the original image.

Serious oversegmentation problems result when the required gradient information is based solely on pixel intensities [23]. To reduce the over-segmentation problem, *texture gradients*, as introduced by Hill et al. [24], are used instead of intensity gradients. Different textures contain information that can be used to identify different tissues. If the gradients between textures are detected and used as input to the watershed algorithm, the images can be segmented into several homogeneous texture regions.

In this paper, the texture gradient is derived from the Dual-Tree Complex Wavelet Transform (DT-CWT) coefficients [24]. DT-CWT calculates the complex wavelet transform of a signal using two separate real wavelet decompositions. The transform retains the useful properties of scale and orientation sensitivity, is approximately shift invariant, and also provides a representation with reduced redundancy. For each scale level, six subbands are produced, orientated at ±15°, ±45°, and ±75°, retaining the detail information of the original image along six different orientations. The texture gradient is derived from the subband features, where *D*_*i*,*θ*_(*x*_1_, *x*_2_) represents the subband oriented along *θ* at the *i*th scale level.

The texture gradient is obtained in several steps. First of all, directional median filtering [18] is used on each subband *D*_*i*,*θ*_(*x*_1_, *x*_2_). Directional median filtering refers to median filtering adapted to the orientation, *θ*, of the subband, *i*. It is implemented as two 1D median filters, *f*_*M*_(*θ*+*π*/2)__ and *f*_*M*_*θ*__, where the neighbourhood of the first filter extends in a line normal to the subband orientation and removes the step response (double edge effect) of the subbands. The second filter, parallel to the subband orientation, removes the noise of the subbands. Considering both scale and orientation, the subband resulting from the filtering is

(10) $$\begin{array}{c} M_{i,\theta }\left(x_{1},x_{2}\right)=f_{M_{\theta }}\left(f_{M_{\left(\theta +\pi /2\right)}}\left(\left|D_{i,\theta }\left(x_{1},x_{2}\right)\right|\right)\right). \end{array}$$

In practice, the size of the median filter is related to the extent of the filter bank impulse response at that level and was chosen as (7 + 2*i*) [18].

After directional median filtering, the new subbands *M*_*i*,*θ*_(*x*_1_, *x*_2_) are passed to the Gaussian derivative function to estimate their gradients and mitigate noise amplification. The magnitude of the texture gradient *G*_Γ_*i*,*θ*__(*x*_1_, *x*_2_) oriented at *θ* at scale level *i* of each subband is given by

(11) $$\begin{array}{c} G_{\Gamma_{i,\theta }}\left(x_{1},x_{2}\right)=\sqrt{{\left(𝔇\cdot \frac{\partial g\left(x_{1},x_{2}\right)}{\partial x_{1}}\right)}^{2}+{\left(𝔇\cdot \frac{\partial g\left(x_{1},x_{2}\right)}{\partial x_{2}}\right)}^{2}}, \end{array}$$

where *𝔇* denotes *M*_*i*,*θ*_(*x*_1_, *x*_2_) and *g*(*x*_1_, *x*_2_) is the Gaussian function. The single texture gradient map, *G*_Γ_(*x*_1_, *x*_2_), required as input to the watershed algorithm, is calculated as a simple weighted sum of magnitudes [18]

(12) $$\begin{array}{c} G_{\Gamma }\left(x_{1},x_{2}\right)=\underset{i,\theta }{\sum }f_{z}\left(w_{i,\theta }\cdot {\hat{G}}_{\Gamma_{i,\theta }}\left(x_{1},x_{2}\right)\right), \end{array}$$

(13) $$\begin{array}{c} {\hat{G}}_{\Gamma_{i,\theta }}\left(x_{1},x_{2}\right)=\frac{G_{\Gamma_{i,\theta }}\left(x_{1},x_{2}\right)}{\underset{x_{1},x_{2}}{\max\;}\left(G_{\Gamma_{i,\theta }}\left(x_{1},x_{2}\right)\right)}, \end{array}$$

(14) $$\begin{array}{c} w_{i,\theta }=\frac{n_{i}}{\sum_{x_{1},x_{2}}{\hat{G}}_{\Gamma_{i,\theta }}{\left(x_{1},x_{2}\right)}^{2}}, \end{array}$$

where *n*_*i*_ is the number of pixels in the subband image at level *i* and *f*_*z*_ is the simple zero insertion interpolation function.

**(b) Modulated Gradient**

After obtaining the texture gradient of the image, a modulated gradient is obtained. The modulated gradient is based on texture activity as described in [24]. Its purpose is to suppress the intensity gradient in textured areas but leave it unmodified in smooth regions. The measure of texture activity is described by

(15) $$\begin{array}{c} f_{\Gamma }\left(x_{1},x_{2}\right)=e^{R_{\text{half}}(E_{\Gamma }(x_{1},x_{2}\;)/\lambda -\psi )} \end{array}$$

where *R*_half_(*ζ*) is half-wave rectification to suppress negative exponents:

(16) $$\begin{array}{c} R_{\text{half}}\left(\zeta \right)=\{\begin{array}{cc} 0, & \text{when}\;\;\zeta <0,\\ \zeta , & \text{when}\;\;\zeta \geq 0. \end{array} \end{array}$$

*λ* and *ψ* are two predefined parameters with values of *λ* = 2 and *ψ* = 7 for any 8-bit grayscale image [18], and the texture energy, *E*_Γ_, is computed from the upsampled subband features which are related to *M*_*i*,*θ*_(*x*_1_, *x*_2_) such that

(17) $$\begin{array}{c} E_{\Gamma }=\underset{i,\theta }{\sum }f_{z}\left(\epsilon_{\kappa }\left(\frac{M_{i,\theta }\left(x_{1},x_{2}\right)}{2^{i}}\right)\right), \end{array}$$

where *ϵ*_*κ*_ is the morphological erosion operator with structure element *κ*. *κ* in this case is a square neighborhood of nine pixels.

**(c) Texture Gradient and Modulated Gradient Combined**

Now, the texture gradient and the modulated gradient are combined to obtain a final “Modified" gradient, *G*_*M*_(*x*_1_, *x*_2_), which captures the perceptual edges in the image

(18) $$\begin{array}{c} G_{M}\left(x_{1},x_{2}\right)=\frac{\left|\nabla F\left(x_{1},x_{2}\right)\right|}{f_{\Gamma }\left(x_{1},x_{2}\right)\cdot \mu_{I}}+\frac{G_{\Gamma }\left(x_{1},x_{2}\right)}{\mu_{T}}, \end{array}$$

where *μ*_*T*_ is the median value of the texture gradient, *μ*_*I*_ is defined to be four times the median intensity gradient, and ∇*F*(*x*_1_, *x*_2_) is the gradient of the original image. Figure 4 gives a good illustration of this process.

As a final step in this stage, the H-minima transform [16] is used as a postprocessing technique to improve the segmentation results by modifying the gradient surface and suppressing shallow minima. Stage I outputs a label map, an image where each segmented region is given a unique label, for use in Stage II.

#### 2.2.2. Stage II: Texture Classification and Feature Extraction

All the methods in the previous section are gradient modifications and provide only a partial solution to the watershed over-segmentation problem in real medical images. A novel texture classification method is used to merge regions of similar textures, thus further reducing the oversegmentation and improving algorithm performance.

Traditional texture classification is based on a rectangular-shaped window of a fixed sized [23]. The traditional method treats the “small” area in the window as a texture and attempts to extract the texture features from it. When the window lies completely inside the region of the texture to be represented, one texture feature is extracted. When the window crosses several regions, the features extracted from the window represent a mixture of textures. Rather than using a fixed window-size, the method in the current research uses the regions from the oversegmented image output from Stage I as a basis for texture extraction [25]. Each of these regions has sufficient and homogenous texture information to allow for feature extraction. The texture in each region is compared to the texture of neighbouring regions. If the textures are “similar,” the regions are merged. Similarity is determined using the Kolmogorov-Smirnov test (KS-test) in the following manner.

The texture feature is extracted from a region using a method that is based on the DT-CWT coefficients, relying on their shift invariance and selective sensitivity. The DT-CWT decomposes an image into seven subband images at each scale level. Only one of the subband images, filtered by the lowpass filter, is the approximation information of the image. The remaining six subbands contain detail information, which includes texture information. For example, for scale level 4, one approximation subband image and 24 detail subbands can be obtained. Since the DT-CWT allows perfect reconstruction, a black image is substituted for the approximation subband image. When the image was reconstructed using the inverse DT-CWT, the result, the texture map, contained most of the texture information, and no approximation information.

After the construction of the texture map, the original image and the texture map, along with the label map output from Stage I, are passed to the KS test. Two similarity matrices are obtained: *S*_*ks*_1__ for the texture map and *S*_*ks*_2__ for the original image. The final similarity map used for the merge process, *S*_*ks*_, is obtained by combining *S*_*ks*_1__ and *S*_*ks*_2__ using the following formula:

(19) $$\begin{array}{c} S_{ks}=S_{ks_{2}}\cdot e^{\left(S_{ks_{1}}-1\right)}, \end{array}$$

where the original image information has the dominant effect and the texture map has a supplementary effect.

**Figure 3:**
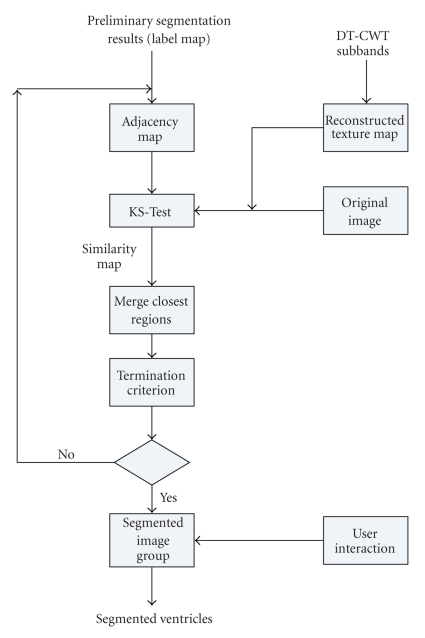
Segmentation algorithm: Stage II.

**Figure 4:**
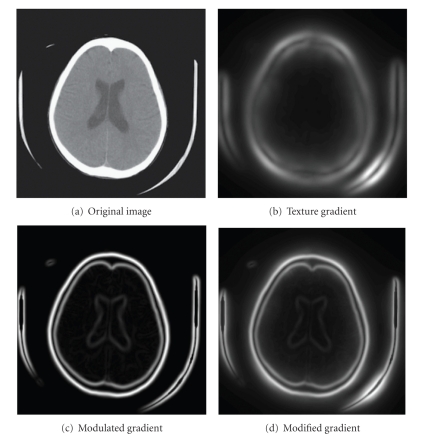
Example of modified gradient for segmentation: Stage I.

The two regions which have the maximum value in *S*_*ks*_ are merged at this step. After merging, the labels for each region are updated and the new segmented image used as input. The flow chart of Stage II is shown in Figure 3. The termination criterion for the “best” segmentation step, determined empirically, is simple. When the maximum value in *S*_*ks*_ equals the minimum value, there are no two regions which should merge.

In summary, an image is oversegmented at the first stage and then a texture classification stage is applied to optimize the outcome of the segmentation until a termination criterion is achieved. Figure 6 shows an example of the final segmentation result obtained from the standard watershed algorithm compared with the result from the adaptive watershed segmentation method used in this research.

#### 2.2.3. User Interactions

Since the watershed segmentation result segments the entire image, and only the ventricles in the image are of interest, some user interactions are included in the framework. This interaction allows the user to identify which regions should be included in the ventricular system. After the regions have been selected, the framework generates an outcome image which only includes the ventricles.

### 2.3. Volume Calculation

The ultimate goal is to calculate the change in the volume of the ventricles. A combination of several algorithms was required to reach this goal. Registration of the two image sets is the first step in this process. Then the ventricles are segmented from the brain tissue. After segmentation, the complete set of slices is used to perform the ventricular volume calculation. The area of the ventricles in each slice is given by

(20) $$\begin{array}{c} a_{k}=p_{s}\cdot p_{s}\cdot n_{v_{k}}, \end{array}$$

where *p*_*s*_ represents the pixel spacing and *n*_*v*_*k*__ the number of pixels in the ventricles in the *k*th slice. The volume of the ventricles in each slice, *V*_*k*_′, is obtained by multiplying the area of the ventricles, *a*_*k*_, by the slice thickness, *τ*_*k*_,

(21) $$\begin{array}{c} V_{k}^{\prime }=a_{k}\cdot \tau_{k}. \end{array}$$

The total volume, *V*′, is obtained by summing the volume of the ventricles in each slice over all the slices which contain the ventricles. The total number of slices which contain ventricle information is represented by *K*

(22) $$\begin{array}{c} V^{\prime }=\overset{K}{\underset{k=1}{\sum }}V_{k}^{\prime }. \end{array}$$

Once the total volume of the ventricles is calculated, the change in volume between registered scans is calculated using (3).

## 3. Data Sets

### 3.1. Physical Phantom

Since it is not possible to measure the true volume of the cerebral ventricles directly in a living person (i.e., without resorting to another image-based morphometric technique), the precision and reliability of the volume calculation framework were tested using a physical phantom with known ventricular volume. A number of physical phantom models have been described in the literature, including plexiglass rods submerged in water cylinders [26] and fluid-filled rubber membranes enclosed in gelatin [8, 9, 27]. In the latter models, the membrane-bound “ventricles” were either of a complex shape [27] or a simple, spherical shape [8, 9], and the fluid was either static [27] or flowing [8, 9]. Models have also included casts of the human ventricular system in formalin-fixed brains [28], potassium iodide baths [29], or copper nitrate baths [26]. These phantoms have either lacked the complex shape of the human ventricular system, required artificial membrane boundaries or used materials that do not mimic the density and texture of brain tissue well on CT. Therefore, in the current research, more realistic agar and water phantoms in a range of sizes were developed for verification and validation of the algorithms.

A set of 5 physical phantoms was constructed [4]. The materials were selected because their densities and textures closely mimic those of real brain tissue and cerebrospinal fluid on CT. Clay models of the human ventricular system, including left and right lateral ventricles, foramina of Munro, third ventricle, cerebral aqueduct, and fourth ventricle, were initially created. These were used to create molds from liquid latex rubber. The molds, in turn, were used to create ice models of the ventricular system, which were immersed in solidifying liquid agar. These phantoms consisting of agar “brain” and water “ventricles” were then scanned, using clinical CT scanning parameters (slice thickness 3 mm at the level of the fourth ventricle and 7 mm above the fourth ventricle, field of view 20 × 20 cm, tube voltage 140 kVp, tube current 140 mAs). Each phantom was given a complete CT scan four times, with the scanning angle changed by 5° between each of the four scans. The volume of water within the phantom's ventricles, *V*_*M*_, was measured using a graduated syringe. The ice model and a sample CT slice image are shown in Figure 7.

### 3.2. Clinical Data

The collection of clinical images was approved by the Research Ethics Board of the IWK Health Centre, and the requirement for informed consent was waived. All clinical CT studies were collected in anonymized DICOM format. The CT studies were from patients whose outcome (normal, stable hydrocephalus, developing hydrocephalus, treated hydrocephalus) was known and were selected by a radiologist (MHS) to reflect a range of outcomes. Of the 13 cases provided, nine cases labeled p*l* were patients who had 2 serial CT scans. The remaining cases labeled p*l* − *m* were patients who had more than 2 serial CT scans. Manual segmentation was also provided by the radiologist (MHS), so that the segmentation portion of the framework could be validated.

## 4. Results

### 4.1. Physical Phantom Results

The volume calculation results for the set of five physical phantoms are summarized in Table 1. The mean calculated volume, $\bar{V^{\prime }}$, refers to the volume calculated by the algorithm framework, averaged over the four scanning angles used. “Mean Error" is the absolute value of the difference between the calculated volume and the measured volume, averaged over the four scanning angles tested and expressed as a percentage. The standard deviation of the calculated volume, *σ*_*V*′_, and of the percentage error, *σ*_*e*_, are noted in the table. The mean percentage error ±1*σ*_*e*_ for all the phantom models was 2.3% ± 0.8%. The maximum percent error for any one volume calculation was 3.5%, therefore the algorithm's margin of error was deemed to be 3.5%.

### 4.2. Clinical Results

#### 4.2.1. Registration Measure

**Figure 5:**
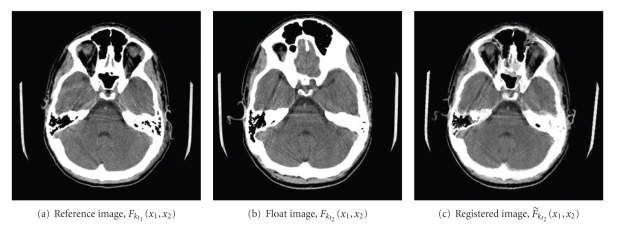
Sample registration result.

**Figure 6:**
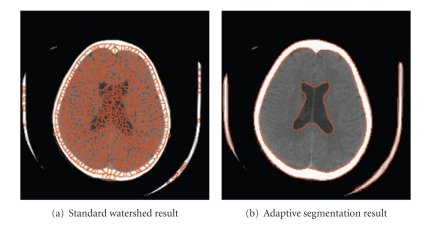
Comparison of segmentation results.

**Figure 7:**
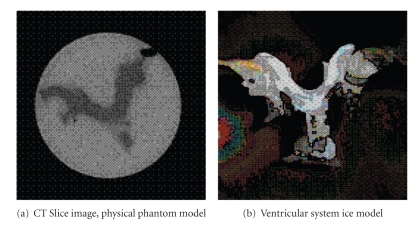
Physical phantom.

The improvement in alignment achieved by the registration algorithm is illustrated in Figure 5. In this example, the 3D displacement of the head between the two CT scans, and its subsequent correction, is particularly noticeable around the eyeballs. In order to quantify the improvement between every image pair, an improvement ratio, *R*, was defined [25]

(23) $$\begin{array}{c} R=\underset{k}{\sum }\frac{\sum_{x_{1},x_{2}}^{}d_{1_{k}}\left(x_{1},x_{2}\right)-\sum_{x_{1},x_{2}}^{}d_{2_{k}}\left(x_{1},x_{2}\right)}{\sum_{x_{1},x_{2}}^{}d_{1_{k}}\left(x_{1},x_{2}\right)}/K, \end{array}$$

where

(24) $$\begin{array}{c} d_{1_{k}}\left(x_{1},x_{2}\right)=\left|F_{k_{t_{1}}}\left(x_{1},x_{2}\right)-F_{k_{t_{2}}}\left(x_{1},x_{2}\right)\right|,\\ d_{2_{k}}\left(x_{1},x_{2}\right)=\left|F_{k_{t_{1}}}\left(x_{1},x_{2}\right)-{\tilde{F}}_{k_{t_{2}}}\left(x_{1},x_{2}\right)\right|. \end{array}$$

The *R* values for all the clinical cases are listed in column 2 of Table 2 and have a mean value of 58.1%. The lowest *R* value, 19.07%, occurred in case p13 − 4 when *F*_*t*_1__ and *F*_*t*_2__ were well aligned before registration. In case p2, with *R* = 20.20%, there was significant skull deformation caused by the hydrocephalus so the registered image, although aligned, is still dissimilar from the initial image.

#### 4.2.2. Segmentation Measure

The segmentation portion of the framework was validated by calculating the similarity index, *S*, between the results of the automated adaptive segmentation and a manual segmentation

(25) $$\begin{array}{c} S=\frac{\sum_{k}^{}S_{k}}{K}, \end{array}$$

(26) $$\begin{array}{c} S_{k}=2\cdot \frac{\left|a_{1}⋂a_{2}\right|}{\left|a_{1}\right|+\left|a_{2}\right|}, \end{array}$$

where *a*_1_ and *a*_2_ are the pixel sets of the ventricle areas, measured in number of pixels, in the images segmented using adaptive segmentation and manual segmentation, respectively. A value of *S* > 0.7 (or 70%) indicates excellent agreement [30]. Table 3 shows the results for each case (ps*i*) with *S* averaged over all the scans in the case as well as over all the slices in the case. *S* ranged from 72.0% to 89.1% with a mean and standard deviation of 76.8% and 5.3%, respectively. The segmentation algorithm worked correctly for cases that had relatively normal ventricles as well as for those that had ventricles enlarged by developing hydrocephalus.

#### 4.2.3. Framework Measure

Since the objective of the research is to measure the change in volume of the ventricular system with time, the difference in volume between two scans was calculated using (3). The change in volume is expressed as a percentage using the following equation:

(27) $$\begin{array}{c} \Delta {\tilde{V}}^{\prime }\text{\%}=\frac{{\tilde{V}}_{t_{2}}^{\prime }-V_{t_{1}}^{\prime }}{V_{t_{1}}^{\prime }}\cdot 100\text{\%}. \end{array}$$

Table 2 summarizes the volume calculation results for all the clinical cases. To further illustrate the effect of registration, the change in volume was calculated both without registration and with it and the results are tabulated as Δ*V*′ and $\Delta {\tilde{V}}^{\prime }$, respectively. By examining the values for Δ*V*′ and $\Delta {\tilde{V}}^{\prime }$, it can be noted that the values generally differ significantly.

**Figure 8:**
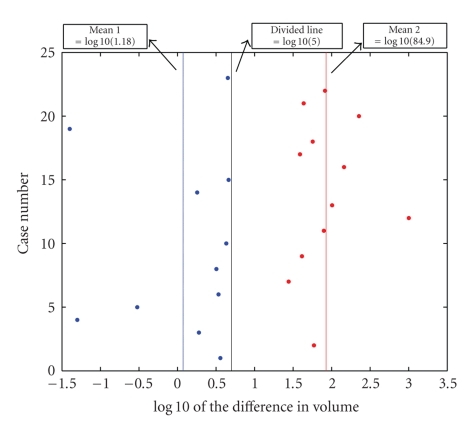
Graphical results for clinical cases: change in volume, $\Delta {\tilde{V}}^{\prime }$, on log scale.

The $\Delta {\tilde{V}}^{\prime }$ values are plotted in Figure 8 on a log scale. This plot shows that the $\Delta {\tilde{V}}^{\prime }$ values separate into two clusters based on k-means clustering of the $\text{lo}{\text{g}}_{10}(|\Delta {\tilde{V}}^{\prime }|)$. The red and blue dots represent the two different clusters. One group has all the $\Delta {\tilde{V}}^{\prime }$ values less than 5% and the other one has the values greater than 20%. A value of $\Delta {\tilde{V}}^{\prime }$ greater than 5% was selected empirically to be the algorithm predictor of developing hydrocephalus. This value was greater than the algorithm's measured accuracy of 3.4% and also allowed a small margin of error for the differences between the physical phantom and the clinical data.

Using this predictor value, the diagnostic performance of the framework was compared to the clinical comments supplied by the radiologist (MHS) and the results are summarized in Table 4 using the following notations.

(TP) true positive: the number of cases which are diagnosed as hydrocephalus and the algorithm output also suggests a hydrocephalus diagnosis.
(TN) true negative: the number of the cases which are diagnosed as nonhydrocephalus and the algorithm also suggests a nonhydrocephalus diagnosis.
(FP) false positive: the cases are non-hydrocephalus but the algorithm suggests a hydrocephalus diagnosis.
(FN) false negative: the algorithm predicts a non-hydrocephalus diagnosis but the true diagnosis is hydrocephalus.

For ease of comparison, the clinical comments associated with each case are also listed in Table 2. The clinical comments were made independently of this research and were supplied by the radiologist (MHS) as a basis for comparison. The following abbreviations are used for these comments: *healthy*: the patient was diagnosed as healthy; *hy*: the patient was developing hydrocephalus; *hy:stable*: the patient has hydrocephalus but the hydrocephalus was stable between the two different scans; *hy:treated*: the patient was diagnosed with hydrocephalus and was treated between scans.

For all the positive and negative examples, the framework prediction and the clinical comments match.

## 5. Conclusion

In this paper, a framework was implemented to measure the volume of the ventricular system to aid in the diagnosis of hydrocephalus. This framework consists of four important algorithms: a modified registration algorithm using a combination of the wavelet multiresolution pyramid and mutual information, an adaptive watershed segmentation with a novel feature extraction method based on the DT-CWT coefficients, and a volume calculation algorithm. In order to quantify the assessment of the success of the algorithms, an improvement ratio was calculated for the registration algorithm and a similarity index for the segmentation algorithm. Finally, physical phantom models with known volumes and clinical cases with known diagnoses were used to verify the volume calculation algorithm. 

The average of *R* for the normal cases is 58.1% indicating that the registration algorithm succeeded in compensating for the displacement between scans. The range of the similarity index for the 13 cases was 72.0% to 89.1% and the average similarity index of all the cases was 76.8% indicating that the segmentation method worked well.

For the volume calculation method on the physical phantom models, all the error rates were below 3.4% and the average error rate was 2.3%, indicating that the accuracy of the algorithm is high. Using $\Delta {\tilde{V}}^{\prime }\geq 5\text{\%}$ as a predictor of developing hydrocephalus, the algorithm prediction matched the clinical comments in all cases. These findings show that the structure of the framework is reasonable and illustrate its potential for development as a tool to aid in the evaluation of hydrocephalus on serial CT scans. 

Future work will include a more rigorous determination of the predictor value as well as collecting and testing a larger set of clinical data to examine the algorithm's performance on a wider range of clinically significant volume changes, particularly small clinically relevant changes.

## Figures and Tables

**Table 1:** Physical phantoms: volume calculation results.

Phantom	*V*_*M*_	$\bar{V^{\prime }}$	*σ*_*V*′_	Mean error	*σ*_*e*_
no.	(cm^3^)	(cm^3^)		(%)	
1	88	89.2	1.3	1.7	0.8
2	101	103.4	1.1	2.4	1.1
3	102	104.4	0.8	2.3	0.8
4	112	115.1	0.8	2.7	0.7
5	132	135.1	0.4	2.3	0.3

	Overall			2.3	0.8

**Table 2:** Volume calculation results for clinical cases.

Case	R	*V*_*t*1_′	*V*_*t*2_′	Δ*V*′	${\tilde{V}}_{t2}^{\prime }$	$\Delta {\tilde{V}}^{\prime }$	Clinical
	%	(cm^3^)	(cm^3^)	(%)	(cm^3^)	(%)	comments
p1	70.9	4.4	4.7	+ 5.6	4.3	− 3.6	healthy
p2	20.2	71.7	169.8	+ 136.9	114.1	+ 59.1	hy
p3	64.2	23.4	24.3	+ 3.9	23.9	+ 1.9	healthy
p4	47.2	4.4	5.6	+ 27.3	4.4	+ 0.1	healthy
p5	62.5	6.7	7.5	+ 12.6	6.7	− 0.3	healthy
p6	63.3	29.8	30.1	+ 1.1	30.8	+ 3.4	hy:stable
p7	55.7	24.1	14.9	− 38.4	17.4	− 27.7	hy:treated
p8	63.8	10.6	12.6	+ 18.8	10.3	− 3.2	healthy
p9-1	53.8	50.1	83.4	+ 66.6	70.6	+ 41.1	hy
p9-2	68.0	83.4	76.9	− 7.8	79.8	− 4.3	hy:stable
p9-3	49.8	76.9	11.1	− 85.6	15.9	− 79.3	hy:treated
p10	67.0	8.5	98.0	+ 1046.9	94.3	+ 1003.7	hy
p11-1	62.0	54.2	149.4	+ 175.8	109.2	+ 101.5	hy
p11-2	55.5	149.4	155.6	+ 4.1	152.1	+ 1.8	hy:stable
p11-3	98.7	155.6	178.5	+ 14.8	162.6	+ 4.6	hy:stable
p12-1	61.2	7.6	21.3	+ 181.6	18.5	+ 144.7	hy
p12-2	58.2	21.3	37.6	+ 77.0	29.5	+ 38.9	hy
p13-1	109.7	42.0	9.8	− 76.6	18.1	− 56.9	hy:treated
p13-2	69.9	9.8	12.5	+ 27.2	9.8	+ 0.04	hy:stable
p13-3	66.4	9.8	39.9	+ 306.3	32.0	+ 226.0	hy
p13-4	19.1	39.9	22.1	− 44.6	22.6	− 43.4	hy:treated
p13-5	41.3	22.1	2.7	− 87.7	3.9	− 82.3	hy:treated
p13-6	41.0	2.7	3.2	+ 16.1	2.8	+ 4.5	hy:stable

**Table 3:** Similarity index calculated between adaptive and manual segmentation.

Case name	Similarity index %
ps1	76.8
ps2	77.1
ps3	72.0
ps4	72.4
ps5	72.4
ps6	74.9
ps7	80.2
ps8	74.6
ps9	72.5
ps10	89.1
ps11	72.4
ps12	80.6
ps13	83.9

Mean	76.8
*σ*	5.3

**Table 4:** Diagnostic performance analysis.

	Predicted positive	Predicted negative	Total
Positive examples	8 (TP)	0 (FN)	8
Negative examples	0 (FP)	5 (TN)	5

Total	8	5	13

## References

[1] Mesiwala AH, Avellino AM, Ellenbogen RG (2002). The diagonal ventricular dimension: a method for predicting shunt malfunction on the basis of changes in ventricular size. *Neurosurgery*.

[2] O’Hayon BB, Drake JM, Ossip MG, Tuli S, Clarke M (1998). Frontal and occipital horn ratio: a linear estimate of ventricular size for multiple imaging modalities in pediatric hydrocephalus. *Pediatric Neurosurgery*.

[3] Synek V, Reuben JR (1976). The ventricular brain ratio using planimetric measurement of EMI scans. *British Journal of Radiology*.

[4] Evans J (2005). *The verification of a computer algorithm designed to calculate the volume of the human cerebral ventricles based on CT
images*.

[5] Huckman MS, Fox J, Topel J (1975). The validity of criteria for the evaluation of cerebral atrophy by computed tomography. *Radiology*.

[6] Fox JH, Jordan LT, Huckman MS (1975). Use of computerized tomography in senile dementia. *Journal of Neurology Neurosurgery and Psychiatry*.

[7] Brann BS, Qualls C, Wells L, Papile LA (1991). Asymmetric growth of the lateral cerebral ventricle in infants with posthemorrhagic ventricular dilation. *Journal of Pediatrics*.

[8] Sze RW, Ghioni V, Weinberger E, Seidel KD, Ellenbogen RG (2003). Rapid computed tomography technique to measure ventricular volumes in the child with suspected ventriculoperitoneal shunt failure I: validation of technique with a dynamic phantom. *Journal of Computer Assisted Tomography*.

[9] Sze RW, Ghioni V, Weinberger E, Seidel KD, Ellenbogen RG (2003). Rapid computed tomography technique to measure ventricular volumes in the child with suspected ventriculoperitoneal shunt failure II: clinical application. *Journal of Computer Assisted Tomography*.

[10] Zatz LM, Jernigan TL (1983). The ventricular-brain ratio on computed tomography scans: validity and proper use. *Psychiatry Research*.

[11] Sun Z (2005). *Using computer vision techniques on CT scans to measure changes in ventricular volume to aid in the diagnosis of
hydrocephalus*.

[12] Auer M, Regitnig P, Holzapfel GA (2005). An automatic nonrigid registration for stained histological sections. *IEEE Transactions on Image Processing*.

[13] Maes F, Vandermeulen D, Suetens P (2003). Medical image registration using mutual information. *Proceedings of the IEEE*.

[14] Pluim JPW, Maintz JBA, Viergever MA (2003). Mutual-information-based registration of medical images: a survey. *IEEE Transactions on Medical Imaging*.

[15] Liu L, Jiang T, Yang J, Zhu C (2006). Fingerprint registration by maximization of mutual information. *IEEE Transactions on Image Processing*.

[16] Soille P (1999). *Morphological Image Analysis, Principles and Applications*.

[17] Shafarenko L, Petrou M, Kittler J (1997). Automatic watershed segmentation of randomly textured color images. *IEEE Transactions on Image Processing*.

[18] O’Callaghan RJ, Bull DR (2005). Combined morphological-spectral unsupervised image segmentation. *IEEE Transactions on Image Processing*.

[19] Maintz JBA, Viergever MA (1998). A survey of medical image registration. *Medical Image Analysis*.

[20] Studholme C, Hill DLG, Hawkes DJ (1999). An overlap invariant entropy measure of 3D medical image alignment. *Pattern Recognition*.

[21] Maes F, Collignon A, Vandermeulen D, Marchal G, Suetens P (1997). Multimodality image registration by maximization of mutual information. *IEEE Transactions on Medical Imaging*.

[22] Pluim JPW, Maintz JBA, Viergever MA (2000). Image registration by maximization of combined mutual information and gradient information. *IEEE Transactions on Medical Imaging*.

[23] Gonzalez RC, Woods RE (2002). *Digital Image Processing*.

[24] Hill PR, Canagarajah CN, Bull DR (2003). Image segmentation using a texture gradient based watershed transform. *IEEE Transactions on Image Processing*.

[25] Luo F (2006). *Wavelet-based registration and segmentation framework for the quantitative evaluation of hydrocephalus*.

[26] Ashtari M, Zito JL, Gold BI, Lieberman JA, Borenstein MT, Herman PG (1990). Computerized volume measurement of brain structure. *Investigative Radiology*.

[27] Brassow F, Baumann K (1978). Volume of brain ventricles in man determined by computer tomography. *Neuroradiology*.

[28] Rottenberg DA, Pentlow KS, Deck MDF, Allen JC (1978). Determination of ventricular volume following metrizamide CT ventriculography. *Neuroradiology*.

[29] Baldy RE, Brindley GS, Ewusi-Mensah I (1986). A fully-automated computer-assisted method of CT brain scan analysis for the measurement of cerebrospinal fluid spaces and brain absorption density. *Neuroradiology*.

[30] Kennedy DN, Filipek PA, Caviness VS (1997). Anatomic segmentation and volumetric calculations in nuclear magnetic resonance imaging. *IEEE Transactions on Medical Imaging*.

